# Characterization of Mercury and Its Risk in Nelson’s, Saltmarsh, and Seaside Sparrows

**DOI:** 10.1371/journal.pone.0044446

**Published:** 2012-09-04

**Authors:** Virginia L. Winder

**Affiliations:** Department of Biology and Marine Biology, University of North Carolina at Wilmington, Wilmington, North Carolina, United States of America; University of Western Australia, Australia

## Abstract

**Background:**

Nelson’s, Saltmarsh, and Seaside Sparrows (*Ammodramus nelsoni*, *A. caudacutus*, and *A. maritimus*, respectively) depend on marsh and wetland habitats – ecosystems in which mercury (Hg) bioavailability is notoriously high. The purpose of the present study was to address the potential impact of Hg on these species using first primary and breast feathers as non-destructive biomonitoring tools.

**Methods and Principal Findings:**

Feathers were sampled from wintering sparrows in North Carolina salt marshes (2006–2010). Feather Hg data were used in three risk analysis components (1) Threshold Component – examined feather Hg with regard to published negative effects thresholds; (2) Hg Dynamics Component – examined Hg in sparrows captured multiple times; and (3) Capture Frequency and Survival Component – tested for links between Hg and return frequency and survival. Threshold Component analyses indicated that Hg concentrations in 42–77% of sampled individuals (breast feather *n* = 879; first primary feather *n* = 663) were within the range associated with decreased reproduction in other avian species. Hg Dynamics Component analyses demonstrated that Hg increased between first and second captures for Nelson’s (*n* = 9) and Seaside Sparrows (*n* = 23). Capture Frequency and Survival Component analyses detected a negative relationship between Hg and capture frequency in Nelson’s Sparrows (*n* = 315). However, MARK models detected no effect of Hg on apparent survival in any species.

**Conclusion and Significance:**

This study indicates that current Hg exposure places a considerable proportion of each population at risk. In particular, 52% of all sampled Saltmarsh Sparrows exhibited first primary feather Hg concentrations exceeding those associated with a >60% reduction in reproductive success in other species. This study reports evidence for net annual bioaccumulation, indicating an increased risk in older individuals. These data can be used to inform future population assessments and management for these species.

## Introduction

Anthropogenic sources account for approximately two-thirds of all input of mercury (Hg) into the environment [1]. Although recent North American restrictions have limited Hg emissions on this continent, global cycling of previous pollution continues, and many areas continue to experience an increase in Hg loading [2]–[4]. Hg biomagnifies (as methylmercury) in both aquatic and terrestrial food webs [5], [6] and can reach concentrations that result in negative effects on wildlife [7], [8]. Because of hydrology, acid-base status and sediment characteristics, marsh and wetland habitats are often areas of high Hg methylation and subsequent bioavailability [9], [10]. As a result, omnivorous songbirds dependent upon these ecosystems can exhibit Hg concentrations comparable to those of piscivorous birds and terrestrial songbirds at point source contaminated sites [5], [11], [12].

Nelson’s, Saltmarsh, and Seaside Sparrows (*Ammodramus nelsoni*, *A. caudacutus*, and *A*. *maritimus*, respectively) co-occur in mixed flocks in North Carolina (NC) salt marshes during their non-breeding season (salt marshes are obligate habitat year-round for the latter two species). Salt marsh habitats in North America are threatened by anthropogenic impacts such as habitat degradation, sea level rise, and pollution [13]. Consequently, each of these species is of conservation concern [14]–[17]. Previous studies have characterized Hg exposure throughout portions of the ranges of Nelson’s and Saltmarsh Sparrows, reporting higher than expected and geographically variable concentrations of Hg in their tissues [11], [12], [18]–[20]. For these reasons, and because of the environmental concerns already identified for these species [13], [21], [22], further research is needed to determine whether Hg concentrations may pose a threat to these populations [23].

In order to assess the impact of Hg exposure on a free-living population, it is necessary to understand several key aspects of that exposure. One such aspect that is commanding the focus of an increasing number of studies is the effect of Hg exposure on reproduction. Two recent studies have provided a solid foundation of reproductive effects threshold data for two avian species, Common Loons (*Gavia immer*) and Carolina Wrens (*Thryothorus ludovicianus*) [7], [8]. The adverse effects levels presented in these two separate studies agree well with one another despite the fact that they were established for very different species. For example, Jackson et al. [8] report 10%, 10–60%, and >60% reductions in nest success when maternal blood Hg concentrations were 0.7, 0.7–2.9, and >2.9 ppm ww, respectively. These blood Hg concentrations are remarkably similar to blood Hg risk categories proposed by Evers et al. [7]: low risk<1.0 ppm ww; moderate risk 1.0–3.0 ppm ww; and high risk >3.0 ppm ww. The similarities in adverse reproductive thresholds between these two studies lend credence to their conservative extrapolation to species for which a species-specific reproductive assessment is not feasible or has not yet been performed.

A second important aspect of Hg exposure involves intra-individual Hg dynamics. Net annual bioaccumulation is reflected in tissue concentrations and occurs when annual intake of a contaminant exceeds an individual’s elimination capacity [7]. This trend has been observed in Common Loons [7] but was not detected in species that experience lower levels of Hg exposure [24]. Understanding Hg dynamics is an important factor in assessing lifetime risk along with the overall health of a population. A third key aspect to understand with respect to Hg exposure is its potential effect on adult survival [23]. The Common Loon is arguably the most thoroughly studied avian species with respect to Hg exposure. However, no clear link between Hg exposure and adult survival has yet been identified for this species [25] despite the fact that reduced reproductive success has been observed at elevated levels of exposure [7], [26]. This disconnect between effects on reproductive success and adult survival highlights our lack of understanding of Hg toxicity and the need for further research to characterize species-specific risks to Hg exposure.

The aim of this study was to use Nelson’s, Saltmarsh, and Seaside Sparrow breast feathers and first primary feathers as non-destructive tools to examine Hg exposure from multiple angles using three related risk analysis components. Each component was designed to increase our understanding of one of the key aspects of Hg exposure described above. The Threshold Component used feather Hg concentrations to determine the proportion of the sampled populations at varying levels of risk based published adverse effects thresholds corresponding to reduced reproductive success for other species [7], [8] (Table 1). Analyses in the Hg Dynamics Component examined intra-individual changes in feather Hg concentrations from year to year, addressing the question of how an individual’s risk changes as it ages. Capture Frequency and Survival Component analyses addressed the question of whether Hg exposure affected capture frequency or survival in the sampled populations.

**Table 1:** Risk categories for tissue Hg based on adverse effects concentrations reported by Evers et al. [7] (Common Loons) and Jackson et al. [8] (Carolina Wrens).

Risk Category	Associated reduction in reproductive success	Body feather Hg (ppm fw)	Flight feather Hg (ppm fw)	Blood Hg (ppm ww)
Low	<10%	<2.4	<3.0	<0.7
Moderate	10–60%	2.4–7.1	3.0–10.4	0.7–2.9
High	>60%	>7.1	>10.4	>2.9

## Results

### Threshold Component

**Figure 1:**
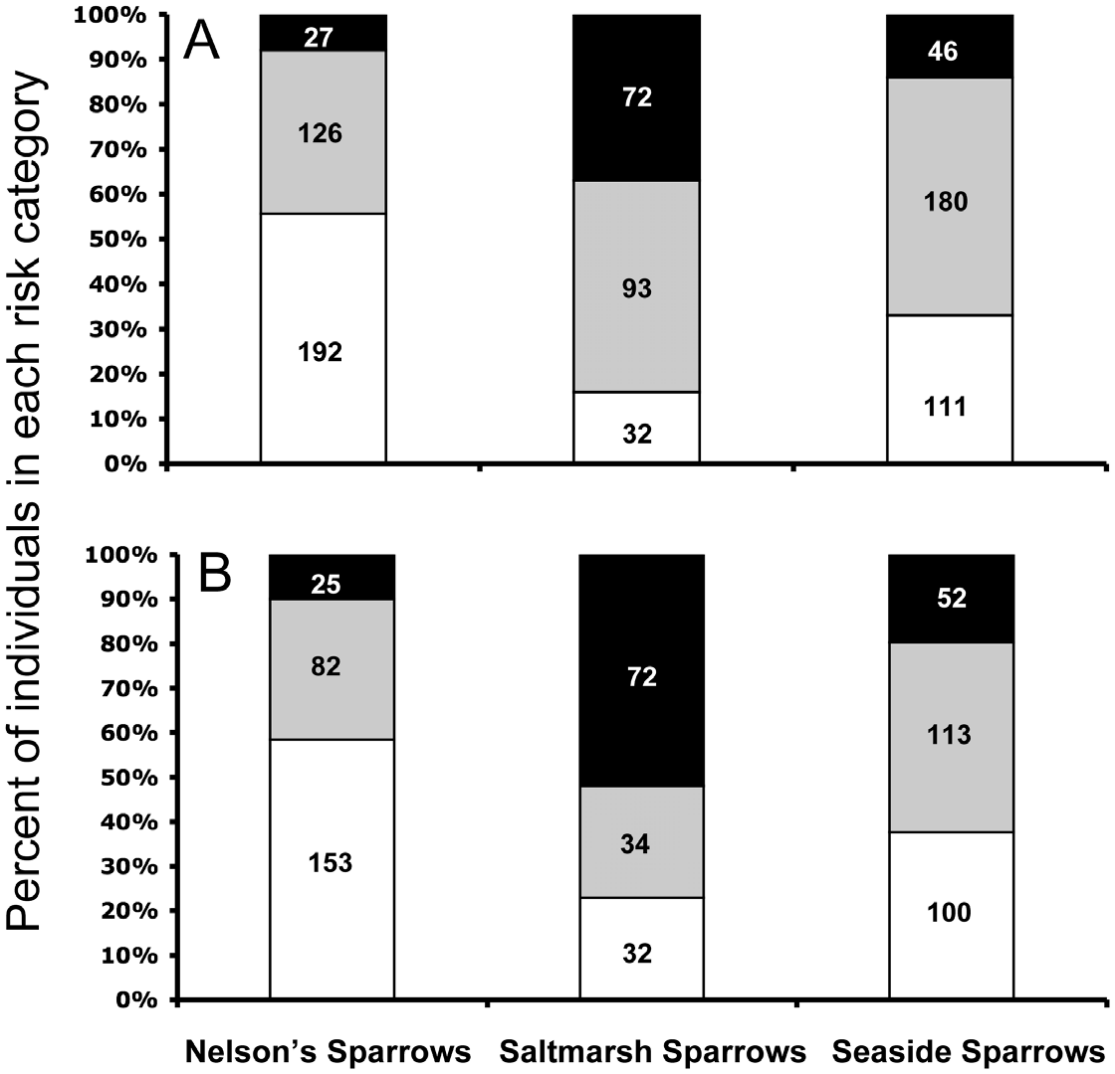
Percent of individuals in each risk category (based on parameters found in **Table 1**). A) Breast feather Hg; and B) First primary feather Hg. Low risk = white, moderate risk = gray, and high risk = black. Numbers inside of bars represent the number of individuals in each category.

An examination of feather Hg concentrations with regard to risk categories (Table 1) revealed that a substantial proportion of the sampled populations is likely at risk due to Hg exposure. For all sampled sparrows, 8–37% were in the high risk category with respect to breast feather Hg; 10–52% were in the high risk category with respect to first primary feather Hg (Fig. 1). Of the three species, Saltmarsh Sparrows exhibit the largest combined proportion of individuals in moderate and high risk categories (84% for breast feathers, 77% for first primary feather), followed by Seaside Sparrows (67% for breast feathers; 63% for first primary feather).

### Hg Dynamics Component

**Figure 2:**
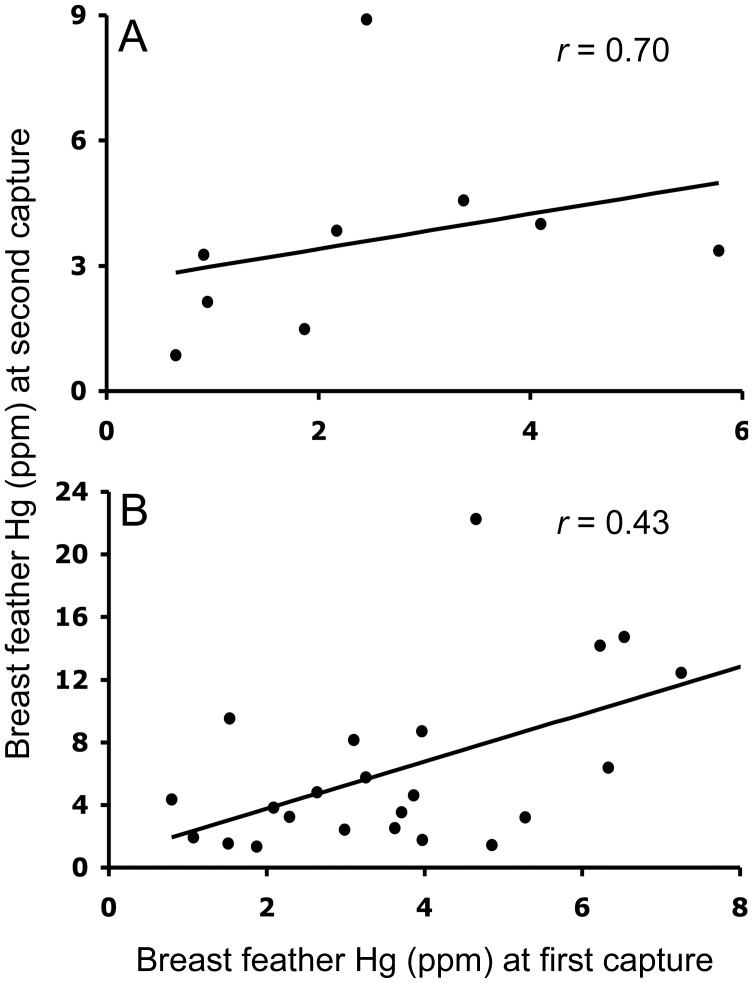
Associations between breast feather Hg at first and second captures (in separate years) for Nelson’s (A) and Seaside Sparrows (B). Linear regression analyses were not applied to these data; lines are for graphical display only.

**Figure 3:**
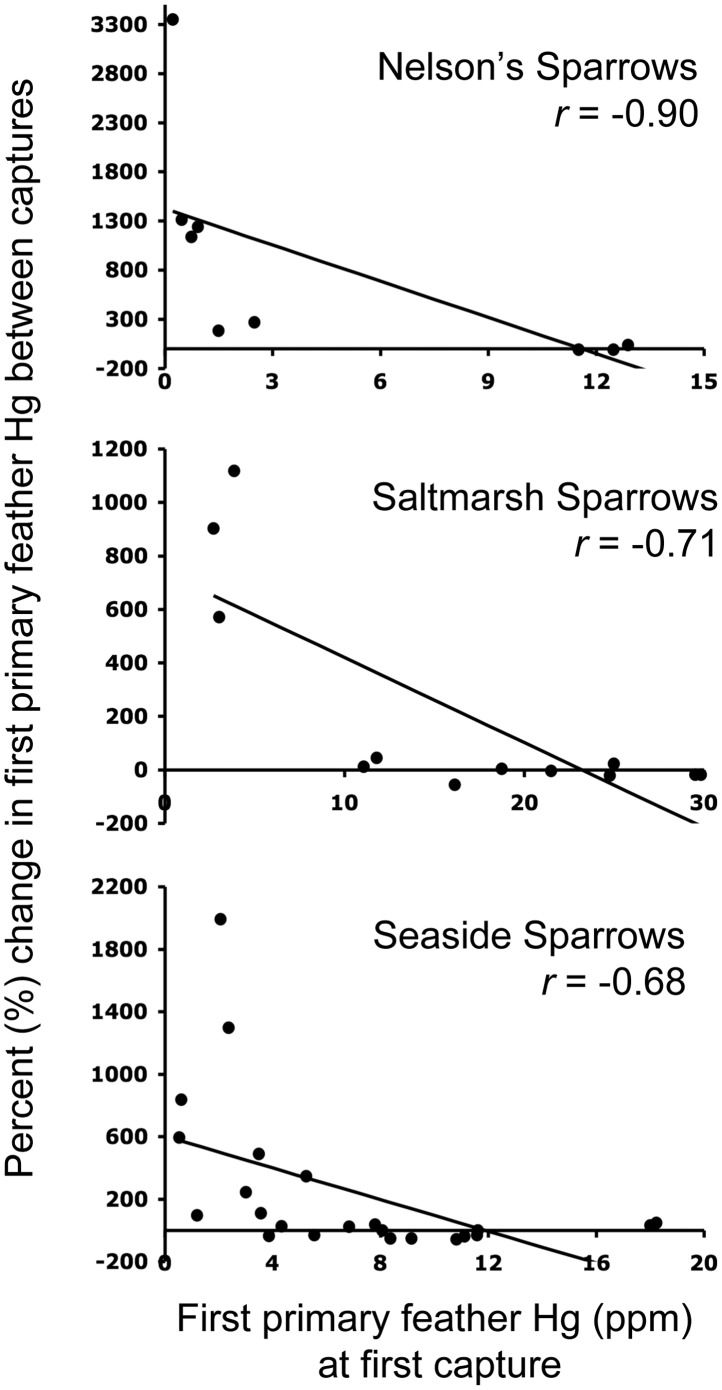
Associations between percent change in first primary feather Hg concentrations between first and second captures (in separate years) and first primary feather Hg at first capture. Linear regression analyses were not applied to these data; lines are for graphical display only.

Hg concentrations in Seaside Sparrow breast feathers were significantly higher (mixed least squares means *P* = 0.0018, *t* = 3.33, *df* = 41, *n* = 23) at second capture (6.07±1.08 ppm) compared to first capture (3.90±0.46 ppm). The same was true for Nelson’s Sparrow primary feather Hg (second capture: 9.79±1.30; first capture: 4.80±1.89 ppm; *P* = 0.0004, *t* = 3.88, *df* = 41, *n* = 9). Spearman correlations revealed a significant positive association between breast feather Hg at first and second capture in Nelson’s Sparrows (*P* = 0.0358, *r* = 0.70, *df* = 8, *n* = 9; Fig. 2A) and Seaside Sparrows (*P* = 0.0386, *r* = 0.43, *df* = 22, *n* = 23; Fig. 2B), but there was no association between these variables in Saltmarsh Sparrows. There was no association between first primary feather Hg at first and second capture or percent change in breast feather Hg between captures and breast feather Hg at first capture for any species. However, Spearman correlations revealed significant negative associations between percent change in first primary feather Hg between captures and first primary feather Hg at first capture for all three species (Nelson’s Sparrows *P* = 0.0009, *r* = −0.90, *df* = 8; Saltmarsh Sparrows *P* = 0.0102, *r* = −0.71, *df* = 11; and Seaside Sparrows *P* = 0.0003, *r* = −0.68, *df* = 22; Fig. 3).

The majority of individuals exhibited an increase in breast feather and first primary feather Hg over time with respect to the 20% change threshold between captures (Table 2). In addition, mean percent increases in feather Hg exceeded mean percent decreases (Table 2). With respect to risk category shifts for breast feather Hg, the majority of individuals (59%) remained within the same risk category between first and second captures, while 36% shifted to a higher risk category at second capture, and 5% shifted to a lower risk category. Patterns for first primary feather Hg were similar to those for breast feather Hg – the majority of individuals (52%) exhibited no change in risk category between captures, 34% shifted to a higher risk category at second capture, and 14% shifted to a lower risk category. Regardless of feather type, there was no difference among species in the occurrence of risk category shifts between captures in different years.

**Table 2:** Number (and %) of individuals exhibiting changes (with respect to the 20% change threshold) and mean changes in feather Hg between captures.

Species	Feather	Individuals increasing (%)	Mean % Increase (±SE) in Hg	Individuals decreasing (%)	Mean % Decrease (±SE) in Hg	Individuals not changing (%)
Nelson’s Sparrow	Breast	6/9 (67)	131 (±43)	2/9 (22)	−31 (±11)	1/9 (11)
	First primary	7/9 (78)	1077 (±430)	0/9 (0)	NA	2/9 (22)
Saltmarsh Sparrow	Breast	5/12 (42)	111 (±25)	3/12 (25)	−33 (±5)	4/12 (33)
	First primary	5/12 (42)	533 (±221)	2/12 (17)	−39 (±17)	5/12 (42)
Seaside Sparrow	Breast	13/23 (57)	178 (±44)	5/23 (22)	−45 (±8)	5/23 (22)
	First primary	14/23 (61)	441 (±156)	7/23 (30)	−42 (±4)	2/23 (9)
Overall	Breast	24/44 (55)	153 (±27)	10/44 (23)	−38 (±5)	10/44 (23)
	First primary	26/44 (59)	630 (±153)	9/44 (16)	−41 (±4)	11/44 (25)

### Capture Frequency and Survival Component

There was a significant effect of breast feather Hg at first capture on capture frequency (multiple [*n* = 26] versus single [*n* = 289]) in Nelson’s Sparrows (proc logistic; *P* = 0.0182, χ^2^ = 5.58, *df* = 1) such that higher breast feather Hg at first capture was associated with birds captured only once (3.20±0.20 ppm) compared to birds captured multiple times (2.06±0.34 ppm). The proportional odds ratio for breast feather Hg from this statistical test was 0.230 and can be interpreted as follows: every 1 ppm increase in breast feather Hg corresponds to 0.230 times greater odds of being captured only once as opposed to multiple times. In other words, a Nelson’s Sparrow with breast feather Hg at 2 ppm is roughly two times more likely to be captured more than once compared to an individual with breast feather Hg at 11 ppm. No effect of breast feather Hg at first capture on capture frequency was observed in Saltmarsh (*P* = 0.4070) or Seaside Sparrows (*P* = 0.2425), and no effect of first primary feather Hg on capture frequency was observed in any species (*P*≥0.1158).

In MARK, the bootstrap goodness-of-fit tests (*P*>0.05) and relatively low variance inflation factors (*ĉ*<5) indicated that the global model met the assumptions of the Cormack-Jolly-Seber (CJS) model. Nine models were excluded from consideration as competitive models on the basis of their inclusion of non-informative parameters [27]–[29]. The most parsimonious model had a 37% chance of being the best candidate model and included the effect of breast feather Hg on apparent survival and the effect of species on capture probability (Table 3). The second-ranked model also included the effect of breast feather Hg on apparent survival and had a 10% chance of being the best candidate model. Eleven additional models had ΔQAIC_c_<7.0 with model weights ranging from 0.10 to 0.01 (Table 3). Breast feather Hg exhibited the highest relative importance of all of parameters with respect to apparent survival with a ∑*w_i_* of 0.59, suggesting that breast feather Hg has some influence on survival. However, the 95% confidence intervals for breast feather Hg beta estimates in each of the 10 models that include this parameter spanned zero, indicating that there is no identifiable trend between breast feather Hg and survival. The relative importance of the effects of species and years on apparent survival resulted in ∑*w_i_* of 0.29 and 0.20, respectively.

**Table 3:** ΔQAIC_c_ rankings for models used to test for the effect of breast feather mercury (Hg) on apparent survival in three species of coastal sparrows in NC salt marshes over five winters.

Model	Δ QAIC_c_	*w_i_*	*k*	QDeviance
Survival (breast feather Hg) Capture (species)	0.000	0.370	7	350.322
Survival (breast feather Hg) Capture (.)	2.657	0.098	5	357.641
Survival (.) Capture (.)	2.686	0.097	4	359.688
Survival (years) Capture (species)	2.687	0.096	9	349.543
Survival (species) Capture (species)	2.771	0.093	8	351.668
Survival (species) Capture (.)	3.095	0.079	6	356.056
Survival (species + breast feather Hg) Capture (.)	3.810	0.055	7	354.742
Survival (breast feather Hg + years) Capture (.)	5.322	0.026	8	354.219
Survival (species + years) Capture (.)	5.983	0.019	9	352.840
Survival (species + years + breast feather Hg) Capture (.)	6.349	0.015	10	351.158
Survival (species + years) Capture (species)	6.428	0.019	11	349.184
Survival (years) Capture (.)	6.460	0.015	7	357.392
Survival (species + breast feather Hg + species × breast feather Hg) Capture (.)	6.720	0.013	9	353.577
Survival (breast feather Hg + years + breast feather Hg × years) Capture (.)	8.212	0.006	11	350.968
Survival (species + years + breast feather Hg + species × breast feather Hg) Capture (.)	9.216	0.004	12	349.914
Survival (species + years + breast feather Hg + species × years) Capture (.)	13.102	0.001	16	345.504
Survival (species + years + species × years) Capture (.)	13.472	0.000	15	347.957
Survival (species + years + breast feather Hg + species × years + species × breast feather Hg) Capture (.)	16.393	0.000	18	344.611

Model notation follows White and Burnham (1999); (_·_) indicates that a given parameter estimate did not vary among species or years; ΔQAIC_c_ indicates quasi-Akaike’s information criterion simple differences adjusted for small sample size and a *ĉ* (variance inflation factor) adjustment of 2.107; *w_i_* indicates Akaike’s model weight; *k* indicates the number of parameters included in a given model. Years refers to the time intervals (between consecutive banding years) for which MARK generates parameter estimates; survival refers to apparent survival; capture refers to capture probability. Models with non-informative parameters were removed from consideration as candidate models and are not shown here.

In order to account for model selection uncertainty in a case where no single model emerged as a clear choice for the best model, I report model-averaged estimates of apparent survival by species and year based on model weights when all 27 candidate models were present in the model set. Overall, model-averaged estimates of survival were similar among species (Table 4). The model-averaged estimate of recapture probability was 0.13±0.05 (unconditional SE) for Nelson’s Sparrows, 0.17±0.07 for Saltmarsh Sparrows, and 0.24±0.08 for Seaside Sparrows.

**Table 4:** Model averaged estimates of apparent survival and accompanying unconditional standard errors for coastal sparrows captured during five winters (2006–2010) in NC salt marshes.

Year^a^	Nelson’s Sparrows		Saltmarsh Sparrows		Seaside Sparrows	
	Apparent survival	SE	Apparent survival	SE	Apparent survival	SE
2006	0.744	0.221	0.712	0.216	0.733	0.200
2007	0.680	0.182	0.650	0.165	0.670	0.148
2008	0.630	0.186	0.601	0.155	0.620	0.143
2009	0.662	0.194	0.634	0.170	0.654	0.153

Model-averaged estimates were calculated based on model weights when all 27 candidate models were present in the model set.

a Refers to year i in survival interval from i to i +1 (e.g. 2006 = interval from 2006 to 2007).

## Discussion

### Threshold Component

Based on published risk categories [7], [8], the sampled Saltmarsh Sparrow population is at the greatest risk to negative effects of Hg of the three study species, with over 75% of Saltmarsh Sparrows falling into either moderate or high risk categories for both first primary feather and breast feather Hg (Fig. 1). A considerable proportion of Nelson’s (>40%) and Seaside Sparrows (>60%) were also placed in either moderate or high risk categories. Based on documented effects in other free-living species, it is reasonable to expect that Hg exposure may be negatively impacting populations of all three study species.

Of the three study species, Saltmarsh Sparrows are also currently of the highest conservation concern [17]. Blood Hg data throughout the breeding range of this species had previously led researchers to speculate that more than a quarter of the global population may be at risk to negative effects [12]. The present study indicates that as much as half of the sampled population of Saltmarsh Sparrows may be at risk to marked reductions in reproductive success due to Hg exposure. This possibility warrants further attention as conservation groups work to assess the global population status of this species in light of other environmental stressors such as habitat loss and degradation and sea level rise.

### Hg Dynamics Component

The risk analyses in the Hg Dynamics Component employed several different methods to characterize changes in Hg over time in recaptured sparrows (Hg dynamics) and relate these to the risk of negative effects due to Hg exposure. I observed an increase in Hg between captures in Nelson’s Sparrows (first primary feather) and Seaside Sparrows (breast feathers). One explanation for this increase is that it is the result of annual Hg intake exceeding elimination – net annual bioaccumulation. Other work with breeding Nelson’s Sparrows has also found evidence for the occurrence of net annual bioaccumulation in this species [30]. One implication of this finding is that as a sparrow ages, it is at greater risk from Hg exposure as its body burden increases year after year. However, in species with a relatively short mean individual lifespan, such an increase in Hg over time may not impact populations to the extent that it would be expected to in a longer-lived species (e.g. Common Loon). A second explanation for the observed association between breast feather Hg at first and second capture is increased Hg availability from year to year, either via increased deposition or methylation. This, combined with strong site fidelity [31], [32] could lead to the pattern observed in this study. This explanation is consistent with the evidence for a temporal increase in Hg exposure on breeding sites for these species during the same time period as the present study [20]. At this time, neither of these explanations can be discounted, and they need not necessarily be considered mutually exclusive.

All three species exhibited a strong, negative association between the percent change in first primary feather Hg and first primary feather Hg at first capture (Fig. 3). Though no age data are available for recaptured individuals in this study, it is possible that individuals with low initial Hg concentrations were hatch-year or relatively young individuals whose Hg body burdens were comparatively low. As these individuals aged, their increasing body burdens represented their bioaccumulation of Hg over time. In contrast, Evers et al. [7] reported that Common Loons with high feather Hg (in excess of 30 ppm) exhibited greater average annual increases compared to the population as a whole (10% increase versus 8.4% year^−1^). This lack of correspondence in feather Hg patterns between loons and sparrows may be related to different physiological processes during feather growth. For example there are extreme differences in flight feather size and molt pattern between Common Loons and sparrows (Common Loons – large feathers, synchronous molt; sparrows – small feathers, sequential molt). In each feather there are a finite number of sulfur-containing amino acids (to which methylmercury can bind). It seems feasible that for a sparrow, with relatively small, sequentially molted flight feathers and a relatively high body burden of Hg for its size, the Hg binding capacity for a given feather could be filled. If, in such a case, net annual bioaccumulation were also occurring, one would expect little to no change in the first primary feather Hg concentration from year to year; however the net annual bioaccumulation would be readily apparent in the Hg concentrations of feathers molted later in the sequence. In Common Loons with large flight feathers molted synchronously (even with very high Hg body burdens), Hg binding capacity in a single feather might never be met, leading to the observed annual increases [7].

Nelson’s and Seaside Sparrows exhibited a positive association between breast feather Hg at first and second captures (Fig. 2). This association is likely caused by consistent dietary patterns and site fidelity. Hg exposure has been documented to vary widely from site to site [11], [12], [33]. If an individual feeds at the same trophic position and returns to the same site consistently year after year, its tissue Hg concentrations would be expected to be associated from one year to the next, reflecting a specific pattern of annual exposure. An understanding of this pattern becomes especially meaningful from a management perspective when particular sites or regions are identified as areas of high Hg availability [11], [30], [33], [34], resulting in Hg concentrations in excess of negative effects thresholds such as were observed in numerous individuals in the present study.

Results from both analyses describing change in feather Hg concentrations across years (as either percent difference with ±20% threshold or shift between risk categories) indicated that an increase in mercury (risk) over time was at least twice (and as many as 8.5 times) as likely as a decrease. In addition, increases in Hg over time tended to be proportionally greater than decreases. The magnitude of the observed changes in Hg between captures far exceeds that reported in adult Common Loons for which feather Hg increased at an average rate of 8.4% year^−1^ [7].

There was an overall paucity of significant associations with regard to Hg dynamics in Saltmarsh Sparrows. For example, feather Hg increased between captures in Nelson’s and Seaside Sparrows but not in Saltmarsh Sparrows. Previous work has documented that Saltmarsh Sparrows consistently exhibit higher Hg concentrations compared to other songbirds at shared sites [12], [18]–[20], but it has yet to be determined whether this disparity is caused by differences in prey selection, physiology, or some other variable. Regardless of the mechanism, comparatively high Hg bioaccumulation in Saltmarsh Sparrows appears to result in chronically high feather Hg concentrations that do not change from year to year and are not related between captures.

### Capture Frequency and Survival Component

In the Capture Frequency and Survival Component, I assessed the effect of Hg on capture frequency and found evidence for effects of Hg exposure unrelated to reproduction in Nelson’s Sparrows. Breast feather Hg concentrations in Nelson’s Sparrows captured only once were, on average, 1.6 times as high as those from the first capture of individuals capture multiple times. Without specific age data for individuals, this study cannot say for certain that the perceived negative effect of Hg on the likelihood of recapture is mutually exclusive from the decreased likelihood of recapture and concomitant increased Hg concentrations with age (see above). In this study, capture frequency depended on a combination of site fidelity, capture probability, as well as survival; it seems feasible that Hg exposure could influence any (or all) of these variables.

The observed link between Hg and capture frequency supports the possibility of a negative effects threshold for Nelson’s Sparrows breast feather Hg concentrations between 2.1 and 3.2 ppm, above which an individual is less likely to be recaptured. Mean breast feather Hg for Nelson’s Sparrows captured multiple times falls within the low risk category while that of Nelson’s Sparrows captured only once falls within the moderate risk category. The correspondence of differential return rates to specific risk categories lends support to the idea that increased Hg concentrations may decrease the likelihood of recapture. However, this study cannot pinpoint the specific cause of reduced return rates for Nelson’s Sparrows with increased breast feather Hg concentrations. As such, it cannot directly implicate Hg as the direct cause of reduced return rates. Both Saltmarsh and Seaside Sparrows exhibited a larger percentage of individuals in the moderate and high risk categories compared to Nelson’s Sparrows, but neither Saltmarsh nor Seaside Sparrows exhibited a relationship between breast feather Hg and capture frequency, indicating that Nelson’s Sparrows may be more sensitive to Hg exposure than the other two species.

MARK analysis of the effect of breast feather Hg on apparent survival rates of Nelsons, Saltmarsh, and Seaside Sparrows indicated that breast feather Hg is the best-supported variable (with respect to an effect on apparent survival) used in the model set. However, this analysis failed to identify a trend between apparent survival estimates and breast feather Hg in these species. A similar study on Common Loons also failed to identify a link between apparent survival and tissue Hg concentrations using MARK analyses [25]. Another similar study on Tree Swallows detected a 1% decrease in apparent adult survival on an Hg-contaminated site (compared to a reference site) using MARK analyses, though the authors speculate that this magnitude of decrease in survival may not be meaningful to population viability in this case [35]. Based on the available comparisons for Common Loons and Tree Swallows, it appears that survival rates may be a less sensitive endpoint with which to assess the effects of Hg exposure compared to reproductive success [7], [25], [35], [36].

I have documented support for a possible link between Hg exposure and capture frequency in Nelson’s Sparrows, but this effect was apparently not robust enough to be detected in the MARK analysis. MARK models may be accurate in not detecting an effect of Hg on survival in these three species, and the observed effect of Hg on Nelson’s Sparrow capture frequency may be spurious. Hg exposure for the species in this study has consistently been documented to be lower at non-breeding sites compared to breeding sites [11], [12], [18]–[20]. Therefore, it seems likely that this reprieve from comparatively high Hg exposure during the breeding season may play a role in lessening the effects of Hg on adult survival [35].

## Materials and Methods

### Ethics Statement

All netting, banding and sampling activities were performed under the requisite institutional, state, provincial and federal permits: University of North Carolina Wilmington Institutional Animal Care and Use Committee 2006–020 and A0910-002, NC Banding Permit 11-BB00039 and Special Research Permit (no associated permit number), and United States Fish and Wildlife Service Master Banding Permit 22935 and Scientific Collecting Permit MB012555-0.

### Study Sites, Capture, and Sampling

Nelson’s, Saltmarsh, and Seaside Sparrows were captured at three sites near Wrightsville Beach, NC (figure 2 in Winder and Emslie 2012) over a period of five winters (2006–2010). Hereafter, I use year to refer to a single non-breeding winter period spanning two calendar years. These relatively elevated salt marsh sites represent areas where sparrows congregate at high tides when much of the surrounding marsh is flooded. Birds were captured by actively funneling individuals from one side of the site toward mist nets. Each individual was banded with a unique U.S. Geological Survey aluminum band. Breast feathers (2006–2010) and the first primary feather (2008–2010) were sampled as described in Winder and Emslie [20].

For the analyses in this study, I used feather Hg data from all individuals captured between 01 November and 31 March in each year to avoid the bias of including transient individuals. Coastal sparrow populations on these NC study sites outside of this time period are comprised primarily of transient individuals moving in and out of the study area during migration [32]. Breast feathers were sampled from 879 individuals (Nelson’s Sparrows *n* = 345, Saltmarsh Sparrows *n* = 197, Seaside Sparrows *n* = 337); first primary feather was sampled from 663 individuals (Nelson’s Sparrows *n* = 260, Saltmarsh Sparrows *n* = 138, Seaside Sparrows *n* = 265). A total of 44 individuals were captured/recaptured and sampled/resampled in multiple years (Nelson’s Sparrows *n* = 9, Saltmarsh Sparrows *n* = 12, Seaside Sparrows *n* = 23). Capture and recapture frequencies were reasonably consistent among years [32].

Feathers were chosen for this analysis because they do not require destructive sampling, allowing the possibility of sampling contamination over multiple years. In addition, feather mercury concentrations represent integrated exposure over an extended time frame, dampening out any short-term changes related to physiological events, which cannot be controlled for in a study of free-living populations. All captured Nelson’s and Saltmarsh Sparrows were winter migrants from unknown breeding locations while captured Seaside Sparrows were likely a mix of year-round NC residents and winter migrants from unknown breeding locations [37]–[39]. Breast feather Hg represents Hg bioaccumulation during the breeding season for each species (regardless of each individual’s specific breeding site) because these feathers are molted biannually [5], [40], [41]. But because specific breeding sites are unknown, conclusions from these data can only be drawn in the broader context of the entire breeding range and seasonal exposure. First primary feather Hg is an index of annual Hg bioaccumulation (integrating exposure throughout the year, again regardless of each individual’s specific breeding site) because these feathers are molted once per year [5], [40], [41]. As such, conclusions from these data are based only on annual exposure.

### Hg Analysis

To remove any externally deposited Hg, feathers were rinsed through three cycles of acetone and deionized water and allowed to dry. Feathers were analyzed for total Hg by atomic absorption spectroscopy using a Milestone® DMA-80 (Shelton, CT, USA) as described in Winder and Emslie [20]. The minimum instrument detection limit during the period of sample analysis ranged from 0.153 to 0.1688 ng; all samples had Hg content above this limit. A method blank, matrix spike (using blood samples from another study), and standard reference material (DOLT-4 or DORM-3 (National Research Council Canada)) were run every 12–20 samples for quality assurance. Recovery of total Hg for standard reference materials ranged from 90–112%, with an average recovery of 100.86±0.58% SE. Matrix spike recovery ranged from 99.4–115.7%, averaging 107.48±0.99% SE.

### Statistical Analyses

All statistical analyses were performed with a significance level at *P*<0.05 using SAS version 9.1 unless otherwise indicated. Data for breast feather Hg and first primary feather Hg met the assumptions for parametric statistical analyses after log_10_ transformation; therefore, log_10_ transformed data were used in statistical tests, but non-transformed values are presented throughout. All results are expressed as arithmetic mean (ppm fresh weight (fw)) ± SE unless otherwise indicated.

#### Threshold Component

To evaluate the risk of wintering coastal sparrows in NC, I used three risk categories based on previous research [7], [8] (Table 1) as reasonably conservative thresholds of possible negative effects with respect to body and flight feather Hg to estimate the risk (linked to reduced reproductive success) of the sampled sparrow populations to Hg exposure. Two logical leaps are required to apply published effects thresholds for Hg concentrations in Carolina Wrens and Common Loons to the data in this study. First, effects concentrations must be extrapolated among species. Based on the high level of agreement between risk categories and associated Hg concentrations in the two studies on very different species described above, it appears that these categories can cautiously be applied to assess possible risks to Hg exposure in other species when species-specific data are unavailable. Second, Jackson et al. [8] present data for Hg concentrations in back and tail feathers while the present study investigated Hg in breast and primary feathers. Since these two pairs of feather types (breast and back; tail and primary) have the same molt pattern within each of these species [41], Hg concentrations within each pair should be representative of the same time frame of exposure such that breast feather Hg is roughly analogous to back feather Hg and likewise for primary and tail feather Hg.

#### Hg Dynamics Component

Analyses in this component were limited to the 44 individuals that were captured/recaptured and sampled/resampled in more than one year. To test whether there was an overall change in breast feather or first primary feather Hg between first and second captures, I used the least squares means output from mixed linear models (proc mixed; model: percent change in Hg between captures = species. I also used Spearman correlations (proc corr) for each species and feather type to test for (1) an association between Hg concentrations at first and second capture; and (2) an association between the percent change in Hg between first and second captures and Hg concentration at first capture.

Two additional metrics were used to examine intra-individual change in Hg across years. In the first, I compared feather Hg concentrations from each individual’s first sampling occasion to those from its first resampling in a subsequent year. I determined the percent change in Hg between captures for breast feather and first primary feather Hg. I considered an individual to have experienced an increase in Hg if the Hg concentration at its second capture was >20% higher than at the first capture, a decrease if feather Hg was >20% lower than at its first capture, and no change if I observed less than a 20% difference between Hg concentrations at first and second captures. A ±20% threshold was used to conservatively allow for intra-individual feather Hg variation and instrument precision. Based on these definitions of increase, decrease and no change, I determined the number of individuals in each category as well as the mean increase and decrease for each species and feather type.

For the second metric, each of the 44 recaptured and resampled individuals was assigned to a risk category (Table 1) based on breast feather and first primary feather Hg at each capture such that a given individual could be placed in the moderate risk category for first primary feather Hg and the low risk category for breast feather Hg. I then determined whether each individual shifted risk categories between first and second captures. To test shifts from one risk category to another varied among species, I used logistic regressions for both breast feather and first primary feather Hg [model: category of change from one risk category to another (with no change as the reference category) = species/link = glogit expb].

#### Capture Frequency and Survival Component

To assess the effect of Hg on capture frequency, I used separate logistic regression models for each species and tissue type to test whether Hg concentrations at the first capture are related to capture frequency (model: capture frequency = Hg at first capture/dist = binomial type3). Capture frequency was constructed as a two-level class variable that was used to separate individuals into two groups – those captured only once and those captured multiple times.

Using mark-recapture data, I built CJS models in MARK [42] to test whether breast feather Hg contributes to variation in apparent survival rates. Breast feather Hg was chosen for this analysis because data for this tissue span five sampling years while those for first primary feather span only three. To estimate apparent survival and capture probabilities, I followed the outline of Lebreton et al. [43]. The capture history of each individual included its presence or absence for five years (2006–2010) with each year composed of banding data from a five-month period (November–March annually). First, I built a global model without covariates, which included species and time parameters [Apparent survival (species + years + species × years) Capture probability (species)]. In MARK models, “years” refers to the time intervals (between consecutive banding years) for which MARK generates parameter estimates. Second, I tested this global model for violations of mark-recapture model assumptions (equal probabilities of catchability and survival) using a bootstrap goodness-of-fit approach, which generated a distribution of expected deviances from the global model using 1,000 random simulations of capture histories. I then calculated the variance inflation factor (a measure of over- or under-dispersion (*ĉ*) by dividing the observed deviance for the global model by the mean expected deviance from the bootstrap simulations, yielding a *ĉ* adjustment of 2.107.

I built 27 additional models, to test a suite of alternate hypotheses incorporating variables that might reasonably influence apparent survival and capture rates. The most parameterized model in the candidate set was structured as follows: [Apparent survival (species + years + breast feather Hg + species × years + species × breast feather Hg) Capture probability (species)]. The remaining 26 models were iterations of this model with one or more parameters removed (Table 3). When an individual had more than one breast feather Hg measurement, I used the mean of its breast feather Hg values to create an index that represents its Hg exposure over the period of its multiple captures. I ranked these 27 models using Akaike’s information criterion (AIC) adjusted for *ĉ* and small sample sizes (QAIC_c_). Models with ΔQAIC_c_<7.0 were considered possible candidates for the most parsimonious model [44]. Models with ΔAIC_c_<2.0 that differed from the best-supported model by only one parameter (or with ΔAIC_c_<4.0 that differed by two parameters, etc.) were excluded from consideration as competitive models on the basis of their inclusion of non-informative parameters [27]–[29]. I examined beta parameter estimates (and accompanying 95% confidence intervals), which can be interpreted as slope parameters representative of the relationship between the covariate and apparent survival. When 95% confidence intervals for a given beta parameter estimate include zero, the data do not support any trend between the covariate and apparent survival.
